# Relationship between reported prior condom use and current self-perceived risk of acquiring HIV among mobile female sex workers in southern India

**DOI:** 10.1186/1471-2458-11-S6-S5

**Published:** 2011-12-29

**Authors:** Anrudh K Jain, Niranjan Saggurti, Bidhubhusan Mahapatra, Mary Philip Sebastian, Hanimi Reddy Modugu, Shiva S Halli, Ravi K Verma

**Affiliations:** 1 Population Council, One Dag Hammarskjold Plaza, New York 10017, USA; 2 Population Council, Lodi Road, New Delhi 110003, India; 3 South Asia Network for Chronic Diseases, Public Health Foundation of India, New Delhi, India; 4 University of Manitoba, Canada; 5 International Center for Research on Women, New Delhi, India

## Abstract

**Background:**

With the evolution of Health Belief Model, risk perception has been identified as one of several core components of public health interventions. While female sex workers (FSWs) in India continue to be at most risk of acquiring and transmitting HIV, little is known about their perception towards risk of acquiring HIV and how this perception depends upon their history of consistent condom use behavior with different type of partners. The objective of this study is to fill this gap in the literature by examining this relationship among mobile FSWs in southern India.

**Methods:**

We analyzed data for 5,413 mobile FSWs from a cross-sectional behavioral survey conducted in 22 districts from four states in southern India. This survey assessed participants’ demographics, condom use in sex with different types of partners, continuation of sex while experiencing STI symptoms, alcohol use before having sex, and self-perceived risk of acquiring HIV. Descriptive analyses and multilevel logistic regression models were used to examine the associations between risky sexual behaviors and self-perceived risk of acquiring HIV; and to understand the geographical differences in HIV risk perception.

**Results:**

Of the total mobile FSWs, only two-fifths (40%) perceived themselves to be at high risk of acquiring HIV; more so in the state of Andhra Pradesh (56%) and less in Maharashtra (17%). FSWs seem to assess their current risk of acquiring HIV primarily on the basis of their past condom use behavior with occasional clients and less on the basis of their past condom use behaviors with regular clients and non-paying partners. Prior inconsistent condom use with occasional clients was independently associated with current perception of high HIV risk (adjusted odds ratio [aOR)] = 2.1, 95% confidence interval [CI]: 1.7-2.6). In contrast, prior inconsistent condom use with non-paying partners was associated with current perception of low HIV risk (aOR= 0.7, 95% CI: 0.5-0.9). The congruence between HIV risk perception and condom use with occasional clients was high: only 12% of FSWs reported inconsistent condom use with occasional clients but perceived themselves to be at low risk of acquiring HIV.

**Conclusion:**

The association between high risk perception of acquiring HIV and inconsistent condom use, especially with regular clients and non-paying partners, has not been completely internalized by this high risk group of mobile FSWs in India. Motivational efforts to prevent HIV should emphasize the importance of accurately assessing an individual’s risk of acquiring HIV based on condom use behavior with all types of partners: occasional and regular clients as well as non-paying partners; and encourage behavior change based on an accurate self-assessment of HIV risk.

## Background

With the evolution of Health Belief Model (HBM) in 1950, risk perception has been identified as one of the several core components of public health interventions, but it is an important marker to promote safe sex behavior in the context of HIV prevention programs [1]. According to this model, individuals must first feel personally threatened by a disease with serious consequences; and then they must believe that the benefits of taking preventive action outweigh the perceived barriers to and/or the cost of preventive action [2,3]. Individuals' knowledge of the modes of HIV transmission and accurate assessment of their own risk seem to be among the key factors in the adoption of safer sexual practices [4].

The relationship between risk perception and sexual behavior is complex and poorly understood in the Indian context. Studies conducted in different cultures and among different populations suggest that HIV risk perception is associated with a wide range of variables: lack of knowledge of the modes of HIV transmission [5-7], young age at first sex [8,9], multiple sexual partners [8,10,11], no or low condom use [8,12,13], not knowing someone with AIDS, and no discussions on AIDS at home [14].

Given the nature of female sex workers’ (FSWs) occupation, reduction in the number of concurrent partners is unlikely to be a practical option to reduce their risk of acquiring HIV. In India, HIV prevention interventions include a strong component of behavioural change communication that attempts to build awareness of HIV risk associated with unprotected sex and the need for consistent condom use to prevent the transmission and acquisition of HIV [1]. In order to support the adoption of safe sex practices, interventions have ensured the availability of condoms [15-18], communicated with sex workers using peers [19-23], made attempts to empower sex workers [18,24-28], provided care for sexually transmitted infections (STIs) and HIV [29,30], and developed collectives and community groups [18,23,27,31,32]. These factors either independently or together have increased the self esteem of sex workers to insist on condom use with their clients [22,24,28,32]. The long-term success of such interventions, however, will depend on the extent to which they bring about sustained change in risky sexual behavior [33,34].

There is little empirical evidence on the role of risky sexual behaviors in predicting the perception of HIV risk among FSWs. While, more than two-thirds of FSWs move from one place to another for sex work and such mobility has been a challenge for HIV prevention programs [35]. The research also shows that FSWs with a higher degree of mobility and short duration of stay in any given place are significantly associated with higher inconsistent condom use with different clients than others [35]. It is not known whether this effect reflects the fact that FSWs who are on the move are less likely to perceive HIV risks in general or are likely to perceive low risk with new clients in new places.

In India, as in many other countries, FSWs are identified as a group at highest risk of acquiring and transmitting HIV, yet little is known about how they, particularly mobile FSWs, themselves perceive their own HIV risk and how their perception depends upon their condom use behaviors with different sexual partners among other factors. The objective of this paper is to assess the HIV risk perceptions of a group of mobile FSWs practicing sex work in four states of India, and to examine the association between reported inconsistent condom use with occasional and regular clients and with non-paying partners, and the high HIV risk perceptions after controlling for several background characteristics and the history/current experience of STI symptoms, and alcohol use before sex. The findings of this assessment will guide the design and modification of communication materials that aim to build an accurate assessment of HIV risk among target populations within extensive HIV prevention programs that are currently underway in India and elsewhere.

## Methods

### Data

This study is based on data from a cross-sectional survey conducted among FSWs from September 2007 to July 2008 in 22 districts from four states in southern (Andhra Pradesh, Karnataka, Maharashtra, and Tamil Nadu) India, identified as high epidemic states by the National AIDS Control Organization (NACO) prior to start of the study in the year 2005. These districts were identified using unpublished mapping and enumeration data on FSWs collected independently by the State AIDS Control Societies and Avahan (the India AIDS Initiative of the Bill & Melinda Gates Foundation). A two-stage sampling procedure was used to select FSWs from both brothel and non-brothel sites. First, small and large sex worker solicitation sites, including brothel areas and open solicitation points such as roads, highways, bus stands, railway stations, and market areas, were mapped. These lists of solicitation sites were used to define and select site clusters, which were formed by combining small areas or by segmenting the large areas such that each cluster included approximately 500 FSWs. Three such clusters from each district were randomly selected, and FSWs were systematically sampled from the brothel areas and from open solicitation points to obtain a minimum of 1,500 eligible participants per state. Eligibility criteria included those aged 18 years or older who had moved to at least two places, one of which included a move across districts, in the past 2 years for sex work. The sample size was determined using an estimated proportion of 30% non-condom use, an assumed difference of 3% increase in the proportion with every unit increase in degree of mobility, a confidence level of 95% and power of 80%.

Across the entire study 9,475 FSWs (out of 10,075 contacted) agreed to be interviewed initially, based on a screening questionnaire. Of these, 59% (n = 5,611) were found eligible according to the eligibility criteria described above. Of the total eligible FSWs (5,611), 198 were excluded: 15 could not be interviewed because they were below age 18 years, 21 refused to participate, 51 withdrew midway without completing the interview, the data on socio-economic variables were missing for 26 FSWs, and 85 FSWs did not answer the question on perceived risk of acquiring HIV. This resulted into a total analytical sample of 5,413 FSWs.

### Ethical procedures

Ethical approval for the study was obtained from the institutional review boards (IRBs) of the Population Council and the University of Manitoba, Canada. Verbal consent was obtained from all respondents prior to participation at each stage. For ethical considerations, only those FSWs who were at least 18 years of age were interviewed.

### Measures

The dependent variable—HIV risk perception—was created from responses to a direct question asked: “What do you think is the risk of your getting HIV?” The response categories included: high, moderate, and low. This information was used to measure whether FSWs perceived themselves to be at high or low risk of acquiring HIV; those who responded as high or moderate risk were coded as 1; and those who responded as low risk were coded as zero. A similar measure of risk perception has also been used in other studies [8,36-38].

Inconsistent condom use was measured for each of the following three types of sex partners: occasional (or new) clients, regular (or repeat) clients, and non-paying partners. For each type of partners, FSWs were asked the number of partners with whom they had sex; the frequency of condom use with these partners (indicated by 1=always, 2=sometimes, 3=never) in the week prior to the survey; and whether or not they had used a condom at last sex. This information was used to create three variables indicating consistent condom use with each type of client: FSWs who reported they had always used condoms in the last week as well as those who had used a condom at last sex were coded as zero (consistent); those who reported they did not have a client of that type in the past week were coded as 2 (no partner); and the rest were coded as 1(inconsistent condom use). The last category included FSWs who reported that they had always used condoms in the last week but had not used a condom at last sex, and those who had sometimes or never used condoms in the last week irrespective of whether or not they had used a condom at last sex.

To determine STI risk, participants were asked whether they had experienced any of the seven symptoms of STIs/reproductive tract infections (RTIs) in the six months prior to the survey and whether they had continued sex while experiencing STI/RTI symptoms. This information was used to create a variable on STI risk: those reported experiencing at least one of the four symptoms (ulcers/sores in genital area, swelling in groin area, pain during intercourse, and frequent painful urination) that are indicative of STI and continued sex were coded as 2; those who experienced STI symptoms but did not have sex were coded as 1; and those who did not experience any STI symptoms in the past six months were coded as zero. Similarly, to determine alcohol use, FSWs who reported “always” consuming alcohol before sex were coded as 1 while the others were coded as zero. Other background characteristics such as age, education, marital status, degree of mobility, living arrangements, currently in debt, experience of sexual violence-- all collected using single questions in the questionnaire were also transformed into binary variables to be used as covariates in the multiple regression analysis.

### Methodological considerations

Two important study variables—condom use behavior and HIV risk perception—are related with each other. This reciprocal (or two way) relationship between the two variables can be viewed as: inconsistent condom use at time 0 —> high HIV risk perception at time 1 —> consistent condom use between time 1 and time 2. These relationships reflect two related questions: (1) does past or recent risky behavior at time 0 explain current high HIV risk perception at time 1, and (2) will current high HIV risk perception at time 1 reduce subsequent risky behavior between time 1 and time 2? While causal inference can adequately be drawn from longitudinal studies [45-47], in the absence of such studies among FSWs in India, we have used cross-sectional data which is more appropriate to address the first question and not the second question [46]. In terms of temporal sequence, explanatory variable should precede the dependent or the outcome variable. We have incorporated the presumed temporal sequence between the two events by considering risky behavior for the period (i.e. condom use in one week prior to survey) preceding the reported self-perceived HIV risk referring to the risk perception at the time of survey.

### Statistical analysis

Bivariate, analysis of variance, and multilevel logistic regression analysis were conducted to study the relationship between reported condom use behavior and self-perceived risk of acquiring HIV. The sample of FSWs in this study is nested within a district which, in turn, is nested within a state. Analysis of variance was conducted to estimate the percent of variance explained by these two cluster variables. Further, to assess the variation existing across these states in terms of number of high risk populations, HIV prevalence and program implementation [30,40-42]; we used a multilevel logistic model for analysis, which also accounted for the design effect. In the multilevel model, the state with four categories was included as the fixed effect and the district with 22 categories as the random effect. Various covariates known to be associated with either condom use behavior or the individual’s perception of HIV risk were included in the multilevel logistic models to eliminate their common effects on the observed relationship between condom use and perceived HIV risk. Adjusted odds ratios were estimated to test these relationships.

Four models were estimated: Model I included the two cluster variables: the district as a random component and the state as the fixed component. These two cluster variables were included in all models. Model II included variables indicating condom use behaviors only; Model III included all the covariates only; and Model IV included condom use variables and other covariates. A comparison of Models II and IV indicates the magnitude of relationship between condom use and perceived HIV risk that is explained by all the covariates included in Model IV. The adjusted odds ratios in Model IV indicate the independent effect of condom use behavior on self-perceived risk of acquiring HIV. All statistical analyses were conducted using STATA version 11.1.

## Results

Of the sample of 5,413 mobile FSWs who were included in this study, 40% currently perceived themselves to be at high risk of HIV. Close to three-fourths (71%) reported consistent condom use in sex with occasional clients in one week prior to the survey; and 60% reported consistent use of condoms in sex with regular clients. About 31% of mobile FSWs reported sex with non-paying partners in the last one week; of these about 57% (or 18% of all FSWs) reported consistent condom use in sex with non-paying partners.

### Association between prior condom use behavior and current perceived high HIV risk

Results presented in Table 1 indicate that the large majority of FSWs who engaged in risky sexual behaviors in last one week also currently perceived themselves to be at a higher risk of acquiring HIV, and that FSWs assessed their HIV risk mainly based on consistency of condom use with occasional clients rather than on consistency of condom use with regular clients or non-paying partners. The effect of reported consistent condom use with regular clients on the degree of perceived risk of acquiring HIV disappears once we control for consistency of condom use with occasional clients. This can be seen by considering FSWs who reported consistent condom use with occasional clients: among these FSWs, the percentage who perceived themselves to be at high HIV risk does not vary by condom use pattern with regular clients (35% among those who reported consistent condom use vs. 39% of those who reported inconsistent condom use with regular clients; panel 1, Table 1). Similarly, among those who reported inconsistent condom use with occasional clients, 56% perceived themselves to be at a high HIV risk among both groups—those reported consistent condom use as well those who reported inconsistent condom use with regular clients. Likewise, the weak relationship between consistency of condom use with non-paying partners and the degree of perceived HIV risk is explained by its relationship with reported consistency of condom use with occasional clients (panel 2, Table 1).

**Table 1:** Percentage of mobile FSWs in Southern India who perceived themselves to be at high risk of acquiring HIV at interview by prior condom use with different type of clients/partners

Condom use in last one week with occasional clients	Condom use in last one week with regular clients				
	
	Consistent	Inconsistent	No client	Total	N
Consistent	34.8	38.9	10.7	33.9	3853
Inconsistent	55.6	55.9	57.0	55.9	1514
No client	28.6	(27.3)	--	28.3	46

Total	35.3	50.9	22.7	40.0	
N	3225	1884	304		5413

**Condom use in last one week with occasional clients**	**Condom use in last one week with non-paying partners**				
	
	Consistent	Inconsistent	No partner	Total	N

Consistent	50.5	53.7	25.2	33.9	3853
Inconsistent	77.4	64.2	52.9	55.9	1514
No client	(83.3)	(33.3)	18.9	28.3	44

Total	51.6	58.7	33.5	40.0	
N	954	714	3745		5413

( ) based on less than 25 FSWs; --- no FSW

These relationships could also be restated in terms of the degree of congruence between prior condom use and self-perceived HIV risk at interview. A high degree of congruence was observed between the degree of self-perceived HIV risk and prior risky behavior with respect to condom use with occasional clients. It appears that about 63% of FSWs were able to assess their HIV risk correctly—47% used condoms consistently and correctly considered themselves to be at low HIV risk, and about 16% of FSWs used condoms inconsistently during past one week and correctly perceived themselves to be at high HIV risk at the time of survey (Table 2). About 36% of FSWs assessed their HIV risk incorrectly: 24% used condoms consistently but perceived themselves to be at high HIV risk, and 12% used condoms inconsistently and perceived themselves to be at low HIV risk.

**Table 2:** Percentage of mobile FSWs according to the degree of congruence between HIV risk perception at interview and prior condom use behavior with occasional clients by states

Congruence between current HIV risk perception and prior condom use with occasional clients	Total	Andhra Pradesh	Karnataka	Maharashtra	Tamil Nadu
***I. Congruent***	**62.6**	**49.9**	**62.3**	**81.1**	**61.3**
a. Consistent condom use and at low HIV risk	47.0	38.4	19.8	77.0	59.8
b. Inconsistent condom use and at high HIV risk	15.7	11.5	42.5	4.1	1.5

***II. Not congruent***	**36.4**	**49.3**	**37.6**	**18.4**	**36.6**
a. Consistent condom use and at high HIV risk	24.1	44.5	5.7	12.3	31.3
b. Inconsistent condom use and at low HIV risk	12.3	4.7	32.0	6.1	5.3

No client	0.8	0.8	0.1	0.4	2.0
Total : %	100	100	100	100	100
N	5413	1533	1420	1188	1272

### State-level differences

Significant differences in HIV risk perception were noted among the four states included in this analysis; the percentage of FSWs who currently perceived themselves to be at a high HIV risk varied from about 17% in Maharashtra to 56% in Andhra Pradesh (Table 3). While the percentage of FSWs who reported consistent condom use with occasional clients in past one week was similar in Andhra Pradesh, Maharashtra, and Tamil Nadu (about 83% to 91%), a higher percent of FSWs in Andhra Pradesh perceived themselves to be at high HIV risk than those in Maharashtra (54% vs. 14%). Only 25% of FSWs in Karnataka reported consistent condom use with occasional clients and 17% with regular clients, which was lowest among all states.

**Table 3:** Percentage of mobile FSWs in India who currently perceived themselves to be at high risk of acquiring HIV by consistent condom use in last one week and states in India

Condom use in last one week	% distribution of FSWs				% currently perceived high HIV risk			
	
	Andhra Pradesh	Karnataka	Maharashtra	Tamil Nadu	Andhra Pradesh	Karnataka	Maharashtra	Tamil Nadu
***Occasional clients***								

Consistent	83.0	25.4	89.3	91.1	53.7	22.2	13.8	34.3
Inconsistent	16.2	74.4	10.3	6.8	71.0	57.0	40.2	21.8
No client	0.8	0.1	0.4	2.0	38.5	50.0	20.0	23.1

***Regular clients***								

Consistent	70.8	17.0	68.6	85.1	53.4	24.0	12.8	36.7
Inconsistent	28.0	78.0	20.2	8.4	64.3	52.9	32.5	17.8
No client	1.2	4.9	11.2	6.5	44.4	57.1	10.5	8.4

***Non-paying partners***								

Consistent	20.9	5.6	5.5	38.5	74.4	15.2	24.6	46.1
Inconsistent	25.1	10.7	11.6	3.1	66.4	54.6	46.4	42.5
No partner	54.1	83.7	82.9	58.3	44.8	49.5	11.8	24.3
Total:%	100.0	100.0	100.0	100.0	56.4	48.2	16.5	33.2
N	1533	1420	1188	1272				

### Multilevel logistic regression analysis

The difference in self-perceived risk of HIV among the states is greater than the difference among districts; clustering by states explains about 9% and by districts explains an additional 4% of the variance in the perceived risk of HIV, i.e., the remaining 87% of the variance in perceived HIV risk is not due to clustering but it is due to individual characteristics. The district effect (the standard deviation for the random effect of districts) reduces from 0.99 to 0.54 once the state is included in the fixed part of the model, and does not change much after individual variables are included. However, the district effect remains significantly greater than zero in all models, which indicates that the difference among districts in the degree of self-perceived HIV risk is not explained by the individual characteristics included in these models. Moreover, district effects within each state indicate that districts in Andhra Pradesh and Karnataka are more homogeneous than those in Tamil Nadu and Maharashtra (data not shown).

Multilevel logistic regression results presented in Table 4 confirm the associations observed in the descriptive analysis, even after controlling for several background characteristics. Inconsistent condom use in past one week with occasional clients was independently associated with currently perceived higher risk of acquiring HIV (adjusted odds ratios [aOR] =2.1, 95% CI: 1.7-2.6). However, inconsistent condom use with regular clients was not independently associated with the level of perceived risk of acquiring HIV (aOR=1.0, 95% CI: 0.8-1.2). Inconsistent condom use, in comparison to consistent condom use, with non-paying partners was associated with lower self-perceived risk of acquiring HIV (aOR=0.7, 95% CI: 0.5 - 0.9). These observed associations between prior consistent condom use behaviors with different types of clients and self-perceived HIV risk at the time of survey are not explained by their common relationships with other covariates: the magnitude of adjusted odds ratios for condom use changed only slightly after controlling for these covariates (Model II vs. Model IV).

**Table 4:** Adjusted odds ratios for current perception of high risk of acquiring HIV among mobile FSWs in Southern India

Characteristics	Model I	Model II	Model III	Model IV
	
	Adjusted OR (95% CI)	Adjusted OR (95% CI)	Adjusted OR (95% CI)	Adjusted OR (95% CI)
**Condom use in past one week with occasional clients**				

Consistent		1		1
Inconsistent		2.85 (2.31 -3.51)		2.07 (1.65 -2.60)
No client		0.84 (0.41 -1.71)		0.95 (0.45 -1.97)

**Condom use in past one week with regular clients**				

Consistent		1		1
Inconsistent		1.16 (0.95 -1.42)		0.99 (0.79 -1.22)
No client		0.92 (0.66 - 1.27)		0.91 (0.64 -1.29)

**Condom use in past one week with non paying partners**				

Consistent		1		1
Inconsistent		0.56 (0.43 -0.72)		0.65 (0.49 -0.86)
No partner		0.32 (0.26 -0.38)		0.52 (0.42 -0.64)

**STI symptoms in last six months & use of condom**				

No STI symptom			1	1
No sex during STI symptom			2.68 (2.31 -3.11)	2.36 (2.02 -2.75)
Continued sex during STI symptom			6.76 (5.56 - 8.23)	5.70 (4.66 - 6.98)

**Used alcohol prior to sex**				

No			1	1
Yes			2.36 (2.04 – 2.71)	2.20 (1.90 – 2.54)

**Reason for entering in to sex work**				

Choice/tradition			1	1
Economic/force			1.39 (1.12 -1.72)	1.39 (1.12 -1.72)

**Sex work**				

Full time			1	1
Part time			1.51 (1.31 -1.75)	1.37 (1.18 -1.59)

**State**				

Tamil Nadu	1	1	1	1
Andhra Pradesh	2.84 (1.43 - 5.66)	2.97 (1.50 -5.89)	1.91 (0.89 - 4.10)	2.16 (1.02 - 4.60)
Karnataka	1.98 (0.99 - 3.93)	1.30 (0.65 - 2.60)	1.59 (0.73 - 3.44)	1.28 (0.59 - 2.78)
Maharashtra	0.37 (0.19 - 0.71)	0.48 (0.25 - 0.93)	0.38 (0.18 - 0.79)	0.45 (0.22 - 0.93)

**Random component**				

District (SD)	0.54 (0.38 - 0.75)	0.54 (0.38 - 0.74)	0.59 (0.42 - 0.83)	0.58 (0.42 - 0.81)
**Regression statistics**				
**Log likelihood**	-3288.778	-3128.85	-2896.539	-2854.597
Districts	22	22	22	22
N	5413	5413	5413	5413

Note:

1. The estimated standard deviation (SD) of the district variable without the state variable was 0.99.

2. Experience of sexual violence, living arrangements, currently in debt, degree of mobility, age, education, and marital status were also included as covariates in Models II and IV. Adjusted odds ratios for these covariates (except age) were not significant at 5% level of significance.

Compared to FSWs who did not experience any STI symptom in last six months, the perception of HIV risk was higher among those who continued to have sex while experiencing STI symptoms (aOR=5.7; 95%CI: 4.7 – 7.0) as well as among those who did not have sex while experiencing STI symptoms (aOR=2.4; 95%CI: 2.0 -2.7). Incorporation of STI symptoms improves the degree of congruence between consistent condom use with occasional clients and self-perceived HIV risk from 63% to 80%. Only 7% of FSWs incorrectly perceived themselves to be at high HIV risk despite of the fact that they used condoms consistently and also did not have any STI symptoms.

Other conditions that may hinder consistent condom use— alcohol use before sex, entered sex work because of economic hardship or force, and engaging in sex work on a part-time basis—were also independently associated with higher perceived risk of acquiring HIV than others. In comparison, such covariates as living alone or with other family members, being in debt at the time of interview, and being relatively more mobile which may hinder the consistent condom use were not independently associated with degree of self-perceived risk of acquiring HIV.

State-level aORs indicate that there is no significant difference among FSWs from Tamil Nadu and Karnataka in terms of the degree of self- perceived HIV risk. However, FSWs from Andhra Pradesh perceive themselves to be at a higher risk than those from Tamil Nadu (aOR = 2.2; 95% CI: 1.0-4.6), and those from Maharashtra perceive themselves to be at a lower HIV risk than those from Tamil Nadu (aOR = 0.5; 95% CI: 0.2-0.9). These state-level effects are independent of the differences due to condom use behaviors and other covariates included in these models.

## Discussion

The current study, based on a cross-sectional behavioral survey of mobile FSWs in four states, documents the high degree of congruence between the reported recent (prior) condom use behavior with occasional clients and self-perceived HIV risk at the time of survey. The association between reported condom use behavior with regular clients or non-paying partners and self-perceived HIV risk is either weak or not significant. These findings indicate that FSWs perceive their risk of acquiring HIV mainly on the basis of whether or not they used condoms consistently with occasional clients rather than condom use behavior with regular clients and non-paying partners. In fact, FSWs who either did not have sex with non-paying partners in one week prior to the survey or used condoms inconsistently with non-paying partners perceived themselves to be at a lower risk of acquiring HIV at the time of survey.

The study findings also show that several other risky behaviors are related to high self-perceived risk of HIV, e.g., experience of STI symptoms in the last six months, continuing sex while experiencing STI symptoms, and the use of alcohol before sex. The observed association between inconsistent condom use with occasional clients and perceived high HIV risk is not explained by their joint relationships with the experience of STI symptoms, alcohol use, and other covariates. Incorporating experience of STI symptoms increases the accuracy of personal HIV risk assessment from 63% to 80%. While STI symptoms have poor specificity among women in general, their experience of such symptoms may indicate the outcome of prior inconsistent condom use. In turn the appearance of STI symptoms among FSWs can be used as a marker for diagnosing and treating STIs as well as reinforcing the message of consistent condom use in all sexual encounters.

Furthermore, the apparent inaccurate perception of high HIV risk among about 7% of FSWs who reported using condoms consistently with occasional clients could simply be a reflection of their perception of high HIV risk associated with their profession. It is possible that this perception has not been modified to low risk with the adoption of consistent condom use. Alternatively, some of these FSWs may not have understood the behavioural communication messages and internalized the links between inconsistent condom use and high HIV risk or may be over-reporting both consistent condom use as well as their HIV risk perception. Nevertheless, the inaccurate perception of high HIV risk by those who reported consistent condom use is not important for controlling the spread of HIV, particularly if they actually used condoms consistently. The critical group of FSWs which should be the focus for controlling the spread of HIV is the 12% who perceived themselves to be at low risk of acquiring HIV even though they reported inconsistent condom use with occasional clients.

However, the finding regarding the congruence between inconsistent condom use during sex with occasional clients and high perceived HIV risk perhaps indicates the success of HIV prevention programs in communicating the HIV risk associated with unprotected sex with occasional clients. This finding is supported by the fact that consistent condom use in sex with occasional clients is high. However, the findings of this study also suggest that education programs may not have adequately emphasized the importance of using condoms consistently in all sexual encounters, especially in sex with regular clients and non-paying partners.

The finding that the variance in the degree of self-perceived HIV risk across districts is not explained by the factors included in the study suggests that there are some important unmeasured individual and district-level contextual factors that have not been included in this study. These may, for example, include the prevalence of STI/HIV and the availability of condoms and STI/HIV treatment in the district, and an individual’s knowledge of peers with STIs, and especially HIV, and knowledge of the probability of HIV transmission during any single unprotected sexual encounter. Differences in these individual and contextual factors would also contribute to the important differences observed among states in the degree of self-perceived risk of HIV. In addition, these state-level differences may reflect differences in the type and nature of sex work and the differential effects of HIV prevention programs, particularly behavioral change communication using IEC materials or peer educators. However, the omission of unmeasured contextual factors at the district and state levels may not be important because these two clustering variables accounted for only 13% of the variance in the self-perceived risk of HIV.

The finding that differences across states are greater than differences across districts may indicate the effect of large variations in HIV prevalence across states. Recent data show that HIV prevalence among FSWs in the southern states of India has begun to decline or stabilize in places where effective interventions have been in place for several years [43]. However, due to differences in intensity and geographic coverage of these interventions, changes in the behaviors of high risk population groups, inconsistent condom use, and HIV prevalence continues to be high in selected districts of some of these states.

The finding that perceived level of HIV risk among FSWs differ by states suggest that the peer education programs in these states have been successful to different degrees, which may itself reflect the differences in the nature of sex work across these states. Therefore, these programs need to modify their message and the content of interaction between peer educators and FSWs. The FSWs from Maharashtra perceive themselves to be at the lowest HIV risk; those from Andhra Pradesh perceive to be at the highest HIV risk; and those from Karnataka and Tamil Nadu are in between the other two states. These differences suggest that the peer education programs in Maharashtra may have been more successful than other states. The emphasis in Karnataka could be on finding ways to enable FSWs to shift from inconsistent to consistent condom use with occasional clients. In Andhra Pradesh and Tamil Nadu, there is a need to reinforce the link between consistent condom use with occasional clients and low HIV risk.

In all the states, there is a need for messages to focus on the importance of using condoms consistently with regular and non-paying partners to reduce the risk of acquiring HIV. The design and success of these interventions in changing risky behavior with regular clients, especially with non-paying partners, would require a better understanding of why FSWs do not use condoms consistently with these partners and why those who do use condoms consistently still perceive themselves to be at a high HIV risk, and why FSWs who do not have non-paying partners perceive themselves to be at lower HIV risk than those who do. It is possible that FSWs do not use condoms with non-paying partners because of unequal power relationships. The current study suggests that FSWs may have emotional and perhaps security stakes in their relationships with non-paying partners, and may perhaps be in denial mode about the risk associated with inconsistent condom use. In-depth studies are needed on how self-assessment of HIV risk could relate to risky behavior with each type of client/partner and how this relationship could vary across different contexts.

While the current cross-sectional study of mobile FSWs has important implications for further research as well as HIV prevention programs, these results should be interpreted with caution because of a few limitations and methodological issues. First, the results of this study cannot be generalized to the non-mobile FSWs without repeating it for a representative sample of all FSWs. Second, answers to questions about consistent condom use may reflect some normative responses and could over-estimate the extent of consistent condom use. However, reported consistent condom use in last one week in this sample of mobile FSWs was lower than those who reported condom use at last sex with each type of client. Furthermore, the extent of this over-estimation of reported consistent condom use with occasional clients may be much lower than that associated with reported consistent condom use with regular clients and non-paying partners.

Third, obtaining accurate information about self-perceived risk is quite challenging. To begin with, risk is a probabilistic concept and it generally indicates the potential or the probability that an action or activity would lead to an undesirable outcome. Risk assessment or an individual’s perception of risk involves an assessment, based on current knowledge and belief. While the life-time consequences of HIV are quite severe, the probability of acquiring HIV with a single unprotected sexual encounter is quite low. However, no attempt was made in this study to explain to the respondents the concept of risk in terms of its probabilistic nature; the data on the reported self-perceived risk of acquiring HIV is based on only one question asked directly. It did not include references to any time period, e.g., the question did not specify whether the risk referred to the present period or to the future, an obvious recommendation from other studies based on review of literature [44,45]. Further, the terms low, moderate, and high risk were not explained to the respondents. Thus, the response “moderate” to the question may reflect some ambivalence in risk perception. Future research of this type should explain the concept of risk to participants and include some time reference for assessed risk as well as a follow-up question to ascertain the respondents’ understanding of the term “moderate” risk. Moreover, questions could also be asked about the source of high or low perceived HIV risk [45], and the perception of risk associated with specific types of risky behavior, e.g., inconsistent condom use with occasional clients, inconsistent condom use with regular clients, and inconsistent condom use with non-paying partners.

Fourth, due to the reciprocal (two way) nature of the relationship between reported risky behavior and perceived HIV risk, these results based on a cross-sectional design do not necessarily imply causation [39,44,46]. While causal inference can adequately be drawn from longitudinal studies, the cross-sectional studies are appropriate to explore the relationship between past risky behavior and current risk perception. The cross-sectional studies are not appropriate to explore the effect of risk perception on subsequent changes in risky behavior [46]. In terms of temporal sequence, explanatory variable should precede the outcome variable and we incorporated the presumed temporal sequence between the two events by considering risky behavior for the period (i.e. condom use in the week prior to the survey) preceding the reported perceived HIV risk at interview. Using this approach for a group of FSWs, we demonstrated the expected positive association between reported recent inconsistent condom use with occasional clients and higher self-perceived current HIV risk [39]. Longitudinal studies are especially important to establish causation between self-perceived high HIV risk at time 1 and reduction in subsequent risky behaviors between time 1 and time 2 [45-47]. Moreover, operations research studies are required to assess the impact of interventions on improvements in the accuracy of perceived HIV risk and its affect on the reduction in subsequent risky behavior.

It should be noted that the relationship between risk perception and the adoption of preventive behaviors is fairly complex. Moreover, sustained preventive behavior requires repeated condom use during each sexual encounter and perception of high risk associated with not using a condom during any sexual encounter. Perceived high risk of acquiring HIV associated with unprotected sex may be necessary but it is not a sufficient condition for the adoption of preventive behavior. For example, many FSWs may charge a higher fee for having unprotected sex even though they may be aware of the risk involved [48]. Many other individual and especially contextual factors may be responsible for hindering or facilitating an individual’s decision to use condoms consistently with a client or a partner. Moreover, overall reduction in risky behavior may also happen with changes in societal norms about safe sex and with the implementation of programs focused on changing these norms and sexual practices at the group level, e.g. 100% condom use in Thailand. Nevertheless, motivational messages to reduce risky behavior should incorporate HIV risk associated with inconsistent condom use with all types of clients and partners.

## Conclusions

This cross-sectional behavioral study of mobile FSWs demonstrates a high degree of accuracy in FSWs’ self-perceived high HIV risk at the time of survey based on their prior condom use behavior with occasional clients. However, the link between condom use behavior with regular clients and non-paying partners and perceived HIV risk is not as clearly understood. Findings of this study have important implications for designing the content of IEC materials and the issues to be discussed by peer educators with FSWs. Specifically, these messages and interactions need to emphasize the importance of using condoms in all sexual encounters not only with occasional clients, but also with regular clients as well as with non-paying partners. Peer educators should also enable FSWs to accurately assess their own risk of acquiring HIV based on such markers as frequency of inconsistent condom use with occasional and regular clients as well as with non-paying partners, experience of STI symptoms, and continuing sex while experiencing STI symptoms.

## List of abbreviations used

AIDS: Acquired immune deficiency syndrome; aOR: Adjusted Odds Ratio; CI: Confidence Interval; FSW: Female Sex Worker; HBM: Health Belief Model; HIV: Human Immunodeficiency Virus; IEC: Information, Education and Communication; IRB: Institutional Review Board; NACO: National AIDS Control Organisation; STI: Sexually Transmitted Infections.

## Competing interests

The authors have no financial benefits or competing interests related to this submitted work.

## Authors' contributions

AKJ led conceptualization, conducted all analyses, and led manuscript development. NS assisted with conceptualization, analytic approach, and manuscript development. BM assisted in manuscript development and literature review. MPS assisted with data analysis. HRM lead fieldwork in two states and helped with interpretation of results. SSH assisted with conceptualization and the interpretation of study findings. RKV assisted with conceptualization of analytic approach, manuscript development, and interpretation of study findings. All authors participated as described above and all read and approved this final submitted manuscript.
